# Association of Body Mass Index with Moderate-Intensity Physical Activity, Eating Behavior, and the Use of the Internet or Games among Korean Middle School Students

**DOI:** 10.3390/children11081000

**Published:** 2024-08-16

**Authors:** Jeonga Kwon, Su-Yeon Roh, Daekeun Kwon

**Affiliations:** 1 Department of Elementary Education, College of First, Korea National University of Education, Cheongju 28173, Republic of Korea; gilddong1234555@knue.ac.kr; 2 Department of Exercise Rehabilitation, Gachon University, Incheon 21936, Republic of Korea; dr.rohpilates@gachon.ac.kr; 3 Institute of Sports Health Science, Sunmoon University, Asan 31460, Republic of Korea

**Keywords:** adolescents, body mass index, eating behavior, internet use, middle school students, moderate-intensity physical activity, playing games

## Abstract

Background/Objectives: This study aimed to investigate the association of body mass index (BMI) with the weekly frequency of moderate-intensity physical activity, eating behavior, and the use of the Internet or games among Korean middle school students. Methods: The data of 23,583 Korean middle school students were collected from the 2023 Korean Student Health Examination. The collected data were analyzed using frequency, chi-square, and multivariate logistic regression analyses. Results: The results also showed that the higher the BMI, the greater the likelihood of moderate-intensity physical activity. The likelihood of breakfast intake reduced as the BMI increased. However, the likelihood of using the Internet or games increased with an increase in BMI. The results revealed that 28% (6594 of 23,583) of middle school students rarely participate in moderate-intensity physical activity, while 32% (7553 of 23,583) participate only once or twice a week. The results also showed that the higher the BMI, the higher the likelihood of moderate-intensity physical activity. Among those who were underweight, the odds ratio (OR) of 3–4 days of participation in moderate-intensity physical activity was 0.764 (95% confidence interval [CI]: 0.664–0.880; *p* < 0.001). Among those who were healthy, the OR of more than 5 days of participation was 1.279 (95% CI: 1.131–1.446; *p* < 0.001). Among those who were overweight, the OR of 3–4 days and more than 5 days of participation was 1.172 (95% CI: 1.019–1.348; *p* = 0.026) and 1.181 (95% CI: 1.011–1.380; *p* = 0.036), respectively. The likelihood of the use of the Internet or games increased with an increase in BMI. The OR of the use of the Internet or games was 0.876 (95% CI: 0.806–0.952; *p* = 0.002) and 0.824 (95% CI: 0.743–0.913; *p* < 0.001) among those who were healthy and those who were overweight, respectively. However, the likelihood of breakfast intake reduced as the BMI increased. Among those who were underweight, the OR of always eating breakfast and mostly eating breakfast was 1.299 (95% CI: 1.114–1.515; *p* < 0.001) and 1.236 (95% CI: 1.045–1.461; *p* = 0.013), respectively. Among those who were healthy, the OR of always eating breakfast was 1.157 (95% CI: 1.026–1.305; *p* = 0.018). Among those who were overweight, the OR of mostly eating breakfast was 1.215 (95% CI: 1.030–1.433; *p* = 0.021). Conclusions: Given this, outdoor sports may increase adolescents’ participation in physical activities by helping them break away from repetitive ball games and increasing their overall interest and participation in physical activities. Overall, our results suggest the need to move away from traditional physical education and develop engaging physical activity programs that motivate students to participate in moderate-intensity physical activities.

## 1. Introduction

Adolescence is a transitional stage before adulthood that brings rapid changes in one’s body and mind. The period of adolescence initiates identity development, and thus, one’s behaviors in adolescence tend to continue into adulthood, thereby establishing one’s health behaviors [1]. Despite this, Korean teenagers tend to neglect their health. They tend to emphasize their studies, resort to eating fast food, and do not exercise regularly. This is likely because Korea’s education system is centered on achieving academic success. This makes it imperative for schools and families to provide health-related knowledge to adolescents and encourage them to apply it in their daily lives, as doing this can form healthy habits. In today’s time, Korean adolescents must engage in regular physical activity, form healthy eating habits, and partake in healthy Internet use to lead a healthy life.

Physical activity improves health outcomes, such as physical fitness, cardiometabolic health (blood pressure, lipid, insulin, and glucose metabolism), bone health, and cognitive function among adolescents [2]. The World Health Organization (WHO) recommends that, on average, adolescents should engage in 60 min of moderate to vigorous physical activity daily [3]. Moderate-intensity physical activity in adolescents has been reported to positively affect their physical and mental health [4]. Specifically, it improves adolescents’ cardiopulmonary function, reduces their risk of obesity, and increases their life satisfaction [5,6,7]. Furthermore, moderate-intensity physical activity, especially during adolescence, lays the foundation for lifelong health and well-being, as it shapes exercise habits and lifelong sports participation.

Despite the benefits of moderate-intensity physical activities, a large number of adolescents worldwide do not meet the recommended criteria [8,9]. In 2019, Guthold et al. [8] reported that 81.0% of adolescents engage in insufficient physical activity based on data from 146 countries/territories. In the age group of 5–19 years, the global prevalence of obesity has increased by approximately eight-fold since 1975, reaching 5.6% in girls and 7.8% in boys [10]. The coronavirus disease 2019 (COVID-19) pandemic has also increased adolescents’ body mass index (BMI). Notably, 30% of the children in North America are overweight or obese [11]. Obesity increases the risk of early puberty in children, menstrual irregularities in adolescent girls, sleep disorders such as obstructive sleep apnea, and cardiovascular risk factors, such as prediabetes, type 2 diabetes, high cholesterol levels, hypertension, non-alcoholic fatty liver disease, and metabolic syndrome [12]. Additionally, children and adolescents with obesity can suffer from psychological issues, such as depression, anxiety, eating disorders, poor self-esteem, body image, and peer relationships [12]. In particular, adolescent obesity negatively impacts adolescents’ bodies and minds and is considered a critical concern worldwide.

Many factors influence obesity in adolescents. Considering that dietary behavior is strongly correlated with body weight, it can be inferred that dietary behavior is strongly related to obesity [13]. One cause of increased adolescent obesity is the consumption of fast food [14]. Fast food refers to convenience food purchased from self-service outlets or carry-out places that do not have wait times [15]. Eating fast food is associated with a higher intake of energy, fat, sodium, added sugar, and sugar-sweetened beverages and a lower intake of fruits, vegetables, fiber, and milk [16]. Consequently, fast food consumption among adolescents causes not only obesity but also other diseases. Frequent consumption of fast foods is accompanied by overweight, abdominal fat gain, impaired insulin and glucose homeostasis, lipid and lipoprotein disorders, systemic inflammation, and oxidative stress [17]. Higher fast-food consumption also increases the risk of diabetes, metabolic syndrome, and cardiovascular disease [17]. Meanwhile, breakfast is considered the most important meal of the day [18]. However, skipping breakfast has become a controversial health issue [19,20]. Many people believe that skipping breakfast can help with weight control [19]. However, studies have reported no significant correlation between skipping breakfast and obesity [21]. Therefore, it is necessary to examine the relationship between breakfast consumption and BMI among Korean adolescents.

As the use and functions of the Internet have increased, there have been rising concerns over excessive Internet use [22]. Internet use duration is closely related to Internet addiction: the longer the duration, the greater the risk of Internet addiction [23]. Consequently, the risk of secondary health problems also increases with excessive Internet use [22]. Currently, there are two perspectives regarding how adolescents play online games. Firstly, understanding how adolescents use the Internet and electronic games is important to encourage usage that contributes positively to their development [24]. Secondly, early detection of abuse or misuse allows for preventive interventions [24]. Notably, some researchers have argued that games can incite violence [24,25], while others have indicated that playing games provides relief from conflicts, fears, and anxieties [25,26]. Regardless, greater time spent on sedentary behavior, such as watching TV, using the computer, and playing a game, is related to higher depressive symptoms, unfavorable body composition, cardiovascular risk factors, poor fitness, lower self-esteem, and lower quality of life [27]. Overall, sedentary behaviors occurring during leisure time may be the most important factors to consider, as these are more consistently associated with health outcomes [27].

This study aimed to investigate the relationship between BMI and moderate-intensity physical activity, eating habits, and the use of the Internet or games among Korean middle school students. Notably, previous studies have shown that the COVID-19 pandemic increased the average BMI of adolescents and the proportion of overweight and obese people [28]. This indicates that adolescents’ health deteriorated during the COVID-19 pandemic. Therefore, the results of this study will contribute to improving Korean middle school students’ quality of life by helping them restore their health and lead healthy lives after the pandemic.

## 2. Materials and Methods

### 2.1. Design and Study Population

This study collected data from the 2023 Korean Student Health Examination survey. The Korean Student Health Examination is an annual survey conducted by the Korean Ministry of Education. The Korean Ministry of Education collects data on the physical development of elementary, middle, and high school students from 1074 schools nationwide and conducts health surveys and check-ups. A total of 87,183 elementary, middle, and high school students participated in the 2023 Korean Student Health Examination. After excluding 63,600 participants who were either not related to the study topic or did not respond, we used the data of 23,583 middle school students. The Korean Ministry of Education sent a questionnaire and a manual to 1074 sample schools. To increase the validity and reliability of the analysis results, heights and weights were measured using a machine by a teacher who received specialized training, and oral examinations were conducted at a dental clinic. In addition, students were specifically instructed on how to participate in the survey and were asked to voluntarily respond to questions related to moderate-intensity physical activity, fast food intake, and breakfast intake. Each school recorded the survey responses from students in the National Education Information System (NEIS) and reported them to the Office of Korean Ministry of Education. The Office of Korean Ministry of Education collected all data using a bottom-up decision-making method, compiled all data sent from each school, and reported it to the Korean Ministry of Education. The Korean Ministry of Education entered all collected data into Excel in Microsoft^®^ Office (Microsoft Corporation, Redmond, WA, USA) and published them on its website. Anyone who wishes to use these data can access them by entering personal information such as gender, age, occupation, and email address. All research procedures were approved by the Korean Ministry of Education (approval number: 112002), and this study was conducted according to the principles outlined in the Declaration of Helsinki.

### 2.2. Measures

#### 2.2.1. Independent Variable

The independent variable was BMI. It was calculated based on participants’ height and weight using the following formula: weight (kg)/(height [m] × height [m]). The results were classified as <18.5 denoting underweight, 18.5–23 denoting normal-weight, 23–25 denoting overweight, and greater than 25 denoting obesity [29].

#### 2.2.2. Dependent Variables

The dependent variables were weekly frequency of moderate-intensity physical activity, eating habits, and the use of the Internet or games. The weekly frequency of moderate-intensity physical activity was measured by asking respondents, “How many days a week do you exercise for 30 min to 1 h in a manner that makes you out of breath or sweat?” The responses were rated on a 4-point Likert scale of 1 (“rarely”), 2 (“1–2 days”), 3 (“3–4 days”), and 4 (“more than 5 days”). We used these responses without any modifications. We identified two variables for eating habits: fast food intake and breakfast intake. Fast food intake was measured by asking respondents, “How many times do you eat fast food in a week?” Responses were rated on a 4-point Likert scale of 1 (“never”), 2 (“1–2 days”), 3 (“3–5 days”), and 4 (“every day”). These responses were used without any modifications. Breakfast intake was measured by asking, “How frequently do you have breakfast?” The responses were rated on a 4-point Likert scale of 1 (“every day”), 2 (“often”), 3 (“rarely”), and 4 (“never”). We used the responses without any modifications. The use of the Internet or games was determined by asking, “Do you spend more than two hours a day on the Internet or playing games?” The responses were rated on a 2-point Likert scale of 1 (“yes”) and 2 (“no”). These responses were also used without any modifications.

#### 2.2.3. Covariate Variables

The covariate variables were sex, sleep, perceived body shape, experience of being bullied, and feelings of unsafety due to violence. Sex was categorized into male and female. The amount of sleep was classified as less than 6 h, 6–7 h, 7–8 h, or more than 8 h. The response options for perceived body shape were very thin, somewhat thin, average, somewhat fat, and very fat. The response options for the experience of bullying and feelings of unsafety due to violence were yes and no. For all covariates, the responses were used without any modifications.

### 2.3. Data Analysis

The collected data were analyzed in the following manner. First, a frequency analysis was conducted to determine the characteristics of the study population. Second, we conducted chi-square analyses to identify significant differences in the characteristics of the study population based on BMI values. Third, we performed multivariate logistic regression analyses to determine the association of BMI with the weekly frequency of moderate-intensity physical activity, eating habits, and the use of the Internet or games. Odds ratios (ORs), 95% confidence intervals (CIs), and p-values were determined. Statistical significance was set at the *p* < 0.05 level. All statistical analyses were performed using SPSS for Windows (version 23.0; IBM Corp., Armonk, NY, USA).

## 3. Results

### 3.1. Characteristics of the Study Population

Table 1 presents the characteristics of the study population. Among the 23,583 individuals, most were male (51.4%). Regarding BMI, normal-weight individuals were the highest in number (44.6%), followed by those who were underweight (24.7%), those who were obese (19.6%), and those who were overweight (11.1%). Most individuals engaged in moderate-intensity physical activity once or twice a week (32.0%) or rarely (28.0%). Most individuals ate fast food once or twice a week (73.8%) or never (16.8%). Most individuals always ate breakfast (41.5%) or on most days (22.8%). Most individuals used the Internet or games (63.6%). Furthermore, individuals who perceived their body shape as average were the highest in number (40.8%).

### 3.2. Differences in the Characteristics of the Study Population Based on BMI

Table 2 presents the results of identifying differences in the characteristics of the study population based on BMI. Sex (χ^2^ = 562.498, *p* < 0.001), weekly frequency of moderate-intensity physical activity (χ^2^ = 146.525, *p* < 0.001), breakfast intake (χ^2^ = 101.071, *p* < 0.001), use of the Internet or games (χ^2^ = 68.929, *p* < 0.001), amount of sleep (χ^2^ = 43.344, *p* < 0.001), perceived body shape (χ^2^ = 12,439.26, *p* < 0.001), and experience of being bullied (χ^2^ = 21.599, *p* < 0.001) differed significantly based on BMI.

### 3.3. Association between BMI and the Weekly Frequency of Moderate-Intensity Physical Activity

Table 3 presents the results of analyzing the association between BMI and the weekly frequency of moderate-intensity physical activity. Among those who were underweight, the OR of 3–4 days of participation in moderate-intensity physical activity was 0.764 (95% CI: 0.664–0.880; *p* < 0.001). Among those who were normal-weight, the OR of more than 5 days of participation in moderate-intensity physical activity was 1.279 (95% CI: 1.131–1.446; *p* < 0.001). Among those who were overweight, the OR of 3–4 days and more than 5 days of participation in moderate-intensity physical activity was 1.172 (95% CI: 1.019–1.348; *p* = 0.026) and 1.181 (95% CI: 1.011–1.380; *p* = 0.036), respectively.

### 3.4. Association between BMI and Breakfast Intake

Table 4 shows the results of the analysis of the association between BMI and breakfast intake. Among those who were underweight, the OR of always eating breakfast and mostly eating breakfast was 1.299 (95% CI: 1.114–1.515; *p* < 0.001) and 1.236 (95% CI: 1.045–1.461; *p* = 0.013), respectively. Among those who were normal-weight, the OR of always eating breakfast was 1.157 (95% CI: 1.026–1.305; *p* = 0.018). Among those who were overweight, the OR of mostly eating breakfast was 1.215 (95% CI: 1.030–1.433; *p* = 0.021).

### 3.5. Association between BMI and the Use of the Internet or Games

Table 5 shows the results of analyzing the association between BMI and the use of the Internet or games. The OR of using the Internet or games was 0.876 (95% CI: 0.806–0.952; *p* = 0.002) and 0.824 (95% CI: 0.743–0.913; *p* < 0.001) among those who were normal-weight and those who were overweight, respectively.

## 4. Discussion

### 4.1. Interpretation of the Findings

We obtained several insightful results. First, the higher the BMI, the higher the likelihood of engaging in moderate-intensity physical activity. This result suggests that overweight and obese middle school students are more likely to engage in moderate-intensity physical activity than underweight ones. However, this finding is in contrast to previous research. For instance, Deforche et al. [30] studied attitudes toward physical activity among normal-weight, overweight, and obese adolescents. In their study, overweight and obese adolescents showed lower sports participation and less positive attitudes toward physical activity. Similarly, Hubbard et al. [31] examined the association between moderate-intensity physical activity and BMI among 517 elementary school students. They found that overweight and obese female students engage in less moderate-intensity physical activity than normal-weight and underweight students. This result partially differs from the findings of the present study [31]. However, the results of these studies can be influenced by factors such as genetics [32], socio-statistical characteristics such as sex and race [33], individual life patterns [34], and persistence in physical activity [35]. Given these discrepancies, further research is warranted.

Studies have suggested that an increase in physical activity leads to a proportional increase in food intake among adolescents [36]. In their study, Liang et al. [37] suggested that physical activity helps reduce the risk of being underweight and overweight but does not help people with obesity. This is because obesity is influenced by both exercise and diet [37]. This suggests that overweight and obese adolescents need to eat breakfast. Therefore, if the moderate-intensity physical activity of middle school students increases with a larger BMI, as shown in the results of this study, the rate of obesity may accelerate further among these students. This implication highlights the need for alternative approaches to managing obesity.

Another result of the present study was that the lower the BMI, the higher the likelihood of breakfast intake. This result suggests that obese middle school students are more likely to skip breakfast than underweight ones. Ma et al. [20] conducted a systematic review and meta-analysis of the association between skipping breakfast and being overweight and obese. They found that skipping breakfast is associated with being overweight and obese, and it increases the risk of being overweight and obesity. Similarly, Saikia et al. [38] found that normal-weight adolescents eat breakfast more regularly compared to overweight and obese adolescents. They also reported that out of 800 adolescents in India, 14.9% of overweight and obese adolescents skip breakfast, while only 4% of normal-weight adolescents skip breakfast. They even claimed that overweight and obese adolescents tend to eat breakfast less regularly and consume more high-calorie snacks when hungry [38]. Overall, it can be concluded that consuming high-calorie snacks instead of breakfast increases the BMI of overweight and obese adolescents.

Our third finding was that the lower the BMI, the lower the likelihood of using the Internet or games. This result indicates that obese middle school students are more likely to use the Internet or games than underweight ones. Supporting this, Canan et al. [39] examined the association between BMI and Internet addiction among Turkish adolescents and found a positive correlation. Meanwhile, Mitchell et al. [40] explored the association between screen time and BMI among adolescents. They found that a longer screen time is associated with increased BMI. These results are partially consistent with our results. These insights can be used to explain the relationship between sedentary behavior and obesity among adolescents. For instance, Stamatakis et al. [41] found that adolescents’ TV viewing duration is highly correlated with their BMI and waist circumference. This correlation highlights the broader implications of a sedentary lifestyle, which is associated with various adverse health effects, including elevated all-cause mortality; CVD mortality; cancer risk; risks for metabolic diseases such as DM, HTN, and dyslipidemia; and musculoskeletal diseases such as knee pain and osteoporosis [42]. In summary, obese adolescents are adopting sedentary behaviors. This sedentary behavior is more likely to lead to internet use, TV watching, and game participation than physical activity, which can lead to health problems such as cancer and metabolic diseases. Overall, these findings suggest that the use of the Internet or games is higher among obese students than among underweight students, which can negatively impact their health.

### 4.2. Practical Implications

As stated earlier, the 2020 WHO recommends that adolescents engage in an average of 60 min of moderate-to-vigorous physical activity daily [3]. However, studies have shown that the participation of adolescents in moderate-intensity physical activity decreases with age [4,8,9]. Meanwhile, our results show that 28% of adolescents do not participate in moderate-intensity physical activity even once a week, and 32% participate only once or twice a week. This decline in physical activity is concerning, as it indicates a gradual progression toward obesity [43]. Consequently, policies and efforts are required to encourage moderate-intensity physical activity among Korean adolescents [44]. Furthermore, it is necessary to move away from traditional methods of physical education and develop engaging physical activity programs.

School sports in Korea tend to include only familiar ball sports, such as soccer, basketball, and volleyball. This emphasis has led to criticism that physical education classes are limited to modern sports [45]. This repetition of modern sports can lead to boredom and fatigue among students and reduce their motivation to participate [46]. Therefore, programs must be developed for different sports to encourage adolescents to participate in physical activities, as positive experiences with diverse physical activities can lead to lifelong sports participation. In other words, outdoor sports should be considered a viable solution to the problem of traditional physical education methods, which could otherwise lead to a crisis in the field.

Jeon et al. [47] surveyed Korean teenagers about their desired activities and found that they prefer sports activities that involve nature, such as camping, hiking, swimming, and surfing. With the introduction of outdoor sports into South Korea’s recently revised 2022 physical education curriculum, physical education teachers should consider outdoor sports when designing physical education programs. Outdoor sports often involve group participation and provide opportunities for personal and social development through challenges, exploration, group problem-solving, and social interaction. Furthermore, they provide the opportunity to escape from everyday life and form new relationships with people outside their school friends [48]. Therefore, outdoor sports may strengthen adolescents’ well-being, self-efficacy, individual empowerment, resilience, and social connectedness [48,49]. These sports may also increase adolescents’ participation in physical activities by making them break away from repetitive ball games and increasing their overall interest and participation in physical activities.

### 4.3. Limitations

This study has some limitations. First, BMI values do not take into account an individual’s muscle mass. Consequently, individuals with a high muscle mass may be categorized as overweight or obese, even if they have relatively low body fat. Additionally, adolescents with a high muscle mass and low body fat may have a high BMI but still be in a healthy state. This study may have overlooked such students. Second, this study was conducted as a secondary study and, thus, had limitations in explaining temporal antecedents or causation. Out of the 87,183 elementary, middle, and high school students who participated in the 2023 Korean Student Health Examination, we only used data from 23,583 middle school students. Moreover, the use of fragmentary inputs of BMI, weekly participation in moderate-intensity physical activity, breakfast intake, and Internet or gaming use restricts our ability to establish causality between variables. Third, the data used in this study were self-reported and not scientifically measured. Particularly, the frequency of moderate-intensity physical activity, fast food intake days, and amount of sleep were self-reported.

## 5. Conclusions

Twenty-eight percent of middle school students do not participate in moderate-intensity physical activity even once a week, and thirty-two percent participate only once or twice a week. Furthermore, the higher the BMI, the more likely middle school students are to participate in moderate-intensity physical activity. These results appear to be influenced by factors such as genetics, socio-statistical characteristics, individual life patterns, and persistence in physical activity. The higher the BMI, the more likely middle school students are to use the Internet or games. Overweight and obese adolescents are more likely to play games, use the Internet, and watch TV because they are sedentary, which negatively impacts their health. It is important to keep overweight and obese adolescents out of a sedentary lifestyle to protect their health. In contrast, the lower the BMI, the more likely middle school students are to consume breakfast. Overweight and obese adolescents should be encouraged to eat a regular breakfast, as they are more likely to gain weight by eating high-calorie foods instead of breakfast. These results highlight the need to deviate from traditional methods of physical education and develop unconventional and engaging physical activity programs that encourage Korean middle school students to participate in moderate-intensity physical activities. Outdoor sports may increase adolescents’ participation in physical activities by helping them break away from repetitive ball games and increasing their overall interest and participation in physical activities.

## Figures and Tables

**Table 1:** Characteristics of the study population (*n* = 23,583).

Characteristic	Categories	*n* (%)
Sex	Male	12,126 (51.4%)
	Female	11,457 (48.6%)
Body mass index	Underweight	5824 (24.7%)
	Normal-weight	10,520 (44.6%)
	Overweight	2613 (11.1%)
	Obesity	4626 (19.6%)
Weekly frequency of moderate-intensity physical activity	Rarely	6594 (28.0%)
	1–2 days	7553 (32.0%)
	3–4 days	5304 (22.5%)
	More than 5 days	4132 (17.5%)
Fast food intake	Never	3970 (16.8%)
	1–2 days	17,395 (73.8%)
	3–5 days	1943 (8.2%)
	Every day	275 (1.2%)
Breakfast intake	Always eating	9775 (41.5%)
	Mostly eating	5372 (22.8%)
	Rarely eating	5219 (22.1%)
	Mostly not eating	3217 (13.6%)
Use of the Internet or games	Yes	15,006 (63.6%)
	No	8577 (36.4%)
Amount of sleep	Less than 6 h	3540 (15.0%)
	6–7 h	9294 (39.4%)
	7–8 h	7830 (33.2%)
	More than 8 h	2919 (12.4%)
Perceived body shape	Very thin	1416 (6.0%)
	Somewhat thin	4292 (18.2%)
	Average	9611 (40.8%)
	Somewhat fat	6334 (26.8%)
	Very fat	1930 (8.2%)
Experience of being bullied	Yes	614 (2.6%)
	No	22,969 (97.4%)
Feelings of unsafety due to violence	Yes	241 (1.0%)
	No	23,342 (99.0%)

**Table 2:** Differences in the characteristics of the study population based on BMI.

Characteristic	Categories	Body Mass Index				χ^2^ (*p*)
		Underweight	Normal-Weight	Overweight	Obesity	
Sex	Male	2703 (46.4%)	4901 (46.6%)	1485 (56.8%)	3037 (65.7%)	562.498 (<0.001 ***)
	Female	3121 (53.6%)	5619 (53.4%)	1128 (43.2%)	1589 (34.3%)	
Weekly frequency of moderate-intensity physical activity	Rarely	1913 (32.8%)	2884 (27.5%)	632 (24.2%)	1165 (25.2%)	146.525 (<0.001 ***)
	1–2 days	1887 (32.4%)	3319 (31.5%)	852 (32.6%)	1495 (32.3%)	
	3–4 days	1118 (19.2%)	2372 (22.5%)	665 (25.4%)	1149 (24.8%)	
	More than 5 days	906 (15.6%)	1945 (18.5%)	464 (17.8%)	817 (17.7%)	
Fast food intake	Never	1010 (17.3%)	1774 (16.9%)	429 (16.4%)	757 (16.4%)	10.956 (0.279)
	1–2 days	4237 (72.8%)	7775 (73.9%)	1959 (75.0%)	3424 (74.0%)	
	3–5 days	507 (8.7%)	841 (8.0%)	194 (7.4%)	401 (8.7%)	
	Every day	70 (1.2%)	130 (1.2%)	31 (1.2%)	44 (0.9%)	
Breakfast intake	Always eating	2649 (45.5%)	4369 (41.5%)	1023 (39.2%)	1734 (37.5%)	101.071 (<0.001 ***)
	Mostly eating	1329 (22.8%)	2380 (22.6%)	627 (24.0%)	1036 (22.4%)	
	Rarely eating	1132 (19.4%)	2314 (22.1%)	617 (23.6%)	1156 (25.0%)	
	Mostly not eating	714 (12.3%)	1457 (13.8%)	346 (13.2%)	700 (15.1%)	
Use of the Internet or games	Yes	3667 (63.0%)	6518 (62.0%)	1637 (62.6%)	3184 (68.8%)	68.929 (<0.001 ***)
	No	2157 (37.0%)	4002 (38.0%)	976 (37.4%)	1442 (31.2%)	
Amount of sleep	Less than 6 h	799 (13.7%)	1663 (15.8%)	366 (14.0%)	712 (15.5%)	43.344 (<0.001 ***)
	6–7 h	2255 (38.7%)	4227 (40.2%)	1043 (39.9%)	1769 (38.2%)	
	7–8 h	1944 (33.4%)	3431 (32.6%)	990 (34.1%)	1565 (33.8%)	
	More than 8 h	826 (14.2%)	1199 (11.4%)	314 (12.0%)	580 (12.5%)	
Perceived body shape	Very thin	1018 (17.5%)	293 (2.8%)	46 (1.7%)	59 (1.3%)	12,439.26 (<0.001 ***)
	Somewhat thin	2511 (43.1%)	1554 (14.8%)	93 (3.6%)	134 (2.8%)	
	Average	1920 (33.0%)	6077 (57.8%)	866 (33.1%)	748 (16.2%)	
	Somewhat fat	300 (5.2%)	2323 (22.1%)	1423 (54.5%)	2288 (49.5%)	
	Very fat	75 (1.2%)	273 (2.5%)	185 (7.1%)	1397 (30.2%)	
Experience of being bullied	Yes	125 (2.1%)	249 (2.4%)	86 (3.3%)	154 (3.3%)	21.599 (<0.001 ***)
	No	5699 (97.9%)	10,271 (97.6%)	2527 (96.7%)	4472 (96.7%)	
Feelings of unsafety due to violence	Yes	53 (0.9%)	114 (1.1%)	24 (0.9%)	50 (1.1%)	1.552 (0.670)
	No	5771 (99.1%)	10,406 (98.9%)	2589 (99.1%)	4576 (98.9%)	

Note: *** *p* < 0.001, assessed using chi-square analyses.

**Table 3:** Association between BMI and the weekly frequency of moderate-intensity physical activity.

Variable		Weekly Frequency of Moderate-Intensity Physical Activity Compared to Rare Participation, OR (95% CI)		
		1–2 Days	3–4 Days	More Than 5 Days
Body mass index	Underweight	0.929 (0.819–1.054) *p* = 0.252	0.764 (0.664–0.880) *p* < 0.001 ***	0.883 (0.756–1.032) *p* = 0.118
	Normal-weight	1.020 (0.921–1.130) *p* = 0.698	1.046 (0.936–1.170) *p* = 0.426	1.279 (1.131–1.446) *p* < 0.001 ***
	Overweight	1.107 (0.971–1.260) *p* = 0.127	1.172 (1.019–1.348) *p* = 0.026 *	1.181 (1.011–1.380) *p* = 0.036 *
	Obesity	1.000	1.000	1.000

Note: OR, odds ratio; CI, confidence interval; * *p* < 0.05, *** *p* < 0.001, assessed using multivariate logistic regression analysis adjusted for amount of sleep, perceived body shape, experience of being bullied, and feelings of unsafety due to violence.

**Table 4:** Association between BMI and breakfast intake.

Variable		Breakfast Intake Compared to Mostly Not Eating Breakfast, OR (95% CI)		
		Always Eating	Mostly Eating	Rarely Eating
Body mass index	Underweight	1.299 (1.114–1.515) *p* < 0.001 ***	1.236 (1.045–1.461) *p* = 0.013 *	1.073 (0.907–1.270) *p* = 0.409
	Normal-weight	1.157 (1.026–1.305) *p* = 0.018 *	1.102 (0.967–1.256) *p* = 0.147	1.043 (0.916–1.188) *p* = 0.526
	Overweight	1.151 (0.987–1.342) *p* = 0.073	1.215 (1.030–1.433)*p* = 0.021 *	1.131 (0.960–1.333) *p* = 0.142
	Obesity	1.000	1.000	1.000

Note: OR, odds ratio; CI, confidence interval; * *p* < 0.05, *** *p* < 0.001, assessed using multivariate logistic regression analysis adjusted for the amount of sleep, perceived body shape, the experience of being bullied, and feelings of unsafety due to violence.

**Table 5:** Association between BMI and the use of the Internet or games.

Variable		Use of the Internet or Games Compared to No Use, OR (95% CI)
Body mass index	Underweight	1.005 (0.906–1.115), *p* = 0.923
	Normal-weight	0.876 (0.806–0.952), *p* = 0.002 **
	Overweight	0.824 (0.743–0.913), *p* < 0.001 ***
	Obesity	1.000

Note: OR, odds ratio; CI, confidence interval; ** *p* < 0.01, *** *p* < 0.001, assessed using multivariate logistic regression analysis adjusted for amount of sleep, perceived body shape, experience of being bullied, and feelings of unsafety due to violence.

## Data Availability

Publicly available datasets were analyzed in this study. These data can be found here: [https://schoolhealth.kr/web/srs/selectSchoolOverview.do]. (accessed on 30 May 2023).
